# Solar H_2_ generation in water with a CuCrO_2_ photocathode modified with an organic dye and molecular Ni catalyst†
†Electronic supplementary information (ESI) available. See DOI: 10.1039/c7sc04476c. Additional data related to this publication is available at the University of Cambridge data repository (https://doi.org/10.17863/CAM.16678).


**DOI:** 10.1039/c7sc04476c

**Published:** 2017-11-27

**Authors:** Charles E. Creissen, Julien Warnan, Erwin Reisner

**Affiliations:** a Christian Doppler Laboratory for Sustainable SynGas Chemistry , Department of Chemistry , Lensfield Road , Cambridge CB2 1EW , UK . Email: reisner@ch.cam.ac.uk

## Abstract

Dye-sensitised photoelectrochemical (DSPEC) cells have emerged in recent years as a route to solar fuel production. However, fuel-forming photocathodes are presently limited by photo-corrodible narrow band gap semiconductors or the small range of available wide bandgap p-type semiconductors such as NiO that display low performance with dyes. Here, we introduce CuCrO_2_ as a suitable p-type semiconductor for visible light-driven H_2_ generation upon co-immobilisation of a phosphonated diketopyrrolopyrrole dye with a Ni-bis(diphosphine) catalyst. The hybrid CuCrO_2_ photocathode displays an early photocurrent onset potential of +0.75 V *vs.* RHE and delivers a photocurrent of 15 μA cm^–2^ at 0.0 V *vs.* RHE in pH 3 aqueous electrolyte solution under UV-filtered simulated solar irradiation. Controlled potential photoelectrolysis at 0.0 V *vs.* RHE shows good stability and yields a Ni catalyst-based turnover number of 126 ± 13 towards H_2_ after 2 h. This precious metal-free system outperforms an analogous NiO|dye/catalyst assembly and therefore highlights the benefits of using CuCrO_2_ as a novel material for DSPEC applications.

## Abstract 2

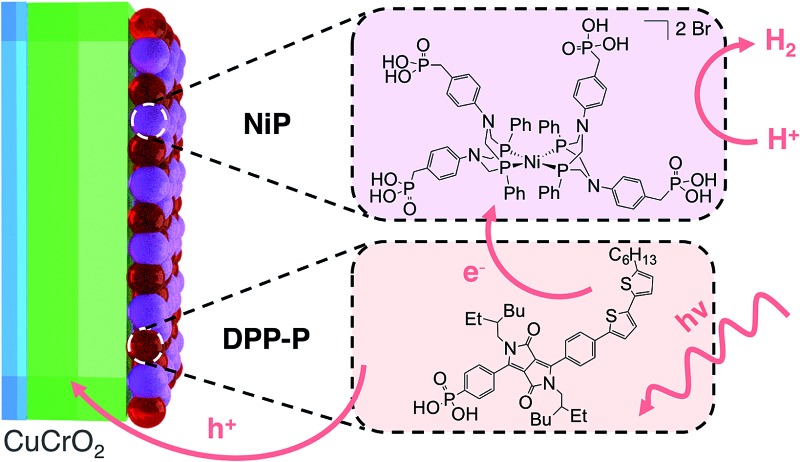
H_2_ generation using a Ni catalyst on dye-sensitised CuCrO_2_ highlights the benefits of using delafossite semiconductors for solar fuel production.

## Introduction

Artificial photosynthesis offers a platform to produce a storable energy supply from fossil fuel-free resources.^1^–^4^ This sustainable, carbon–neutral approach can produce a ‘solar fuel’ such as H_2_ or carbon-based molecules from water or CO_2_ using solar light. This process can be realised using semiconductor electrodes modified with suitable electrocatalysts in a photoelectrochemical (PEC) cell.^5^–^9^ Electrodes featuring a molecular catalyst have advantages over ‘conventional’ heterogeneous systems as their ‘single site catalysis’ is atom-efficient,^10^,^11^ they offer tunability and selectivity for challenging chemical transformations,^12^–^15^ and can be rationally designed to enhance activity.^16^–^19^ Their molecular nature also enables kinetic and mechanistic studies to reveal how they operate under various conditions, outlining routes to improvement.^20^–^23^ Despite these advantages, the development of molecular-based photocathodes is held back by severe material limitations as state-of-the-art electrodes currently lack the requirements of visible light absorption, mesoporosity, p-type conductivity, and/or stability in aqueous solution.^5^,^24^–^27^

To bypass these limitations, a modular approach can be adopted where a visible light-absorbing dye and a molecular catalyst are co-anchored to a stable wide bandgap semiconductor platform.^7^,^28^–^30^ In this dye-sensitised photoelectrochemical (DSPEC) system, the p-type semiconductor serves as the anchor site for the dye, which typically permits ultra-fast hole extraction following visible light excitation of the dye and minimises energy loss. The photoreduced dye is subsequently responsible for electron transfer to the co-immobilised electrocatalyst, where the reduction half-reaction takes place. The separation of light harvesting, charge transport, and catalysis allows the components to be individually tuned for optimal performance, where the rate of each transfer step influences the overall device efficiency.^30^ A suitable pair of photoelectrodes in a tandem DSPEC cell could provide an efficient and inexpensive means of solar fuel production, exploiting simple and adaptable preparation techniques.^31^–^35^

The requirements for a robust DSPEC photocathode material are high p-type conductivity, propensity to anchor molecular moieties, high surface area, and a valence band position capable of readily accepting a hole from the photoexcited dye.^29^,^30^,^36^ Several DSPEC photocathodes have already been reported with the majority relying on NiO,^18^,^37^–^43^ and the only other examples being modified ITO^44^ and CuGaO_2_.^34^ NiO is stable and easily synthesised in mesoporous form,^45^–^47^ but suffers from the drawbacks of low charge carrier mobility and fast charge recombination between valence band holes and the reduced dye.^28^,^48^–^50^ Despite many efforts and different approaches to enhance the PEC properties of dye-sensitised NiO photoelectrodes,^51^–^53^ improvements in performance are hindered by these limitations and there is a crucial need for better alternatives.

Wide bandgap Cu(i)-based mixed metal oxides such as Cu^I^M^III^O_2_ delafossites (M = Co, B, In, Sc, Cr, Al, Ga) have been employed in p-type dye-sensitised solar cells (p-DSSCs),^54^,^55^ but their incorporation in solar fuel devices is limited.^56^–^58^ The sole example of their use with a co-immobilised dye and molecular catalyst in solar fuel generation was reported for CO_2_ reduction to CO with an anchored precious metal-based **Ru–Re** dyad on a CuGaO_2_ delafossite electrode.^34^ Delafossite CuCrO_2_ has shown promise in p-DSSCs but application has yet to be extended to DSPEC cells despite it showing clear benefits such as a low-lying valence band, high hole mobility, and simple and scalable synthesis.^59^–^63^

**Fig. 1:**
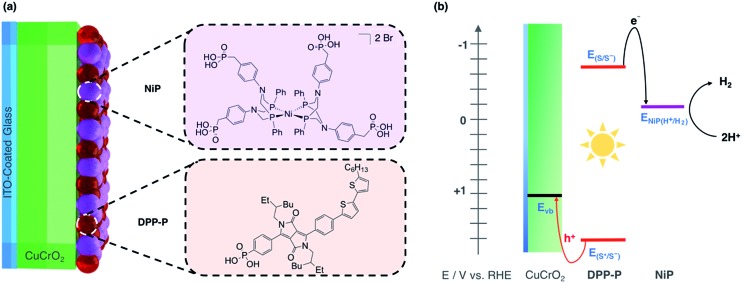
(a) Dye (**DPP-P**) and catalyst (**NiP**) co-immobilised on the CuCrO_2_ electrode with their molecular structures. (b) Energy diagram showing movement of electrons with black and holes with red arrows. S represents the dye sensitiser where *E*_(S/S^–^)_ is the ground state reduction potential and *E*_(S*/S^–^)_ is the reduction potential in the excited state. *E*_NiP(H^+^/H_2_)_ is the catalytic onset potential for **NiP** and *E*_vb_ is the CuCrO_2_ valence band potential.

In this study, we report solar H_2_ generation with dye-sensitised CuCrO_2_ and demonstrate the feasibility of solar fuel synthesis with a CuMO_2_ delafossite using precious metal-free dye/catalyst molecules. This was achieved by first modifying CuCrO_2_ with a phosphonic acid-bearing diketopyrrolopyrrole-based organic dye (**DPP-P**) and characterising the PEC reduction of a soluble electron acceptor in aqueous conditions. Then, a tetraphosphonated Ni-bis(diphosphine), [Ni(P_2_N_2_)_2_]^2+^, molecular catalyst (**NiP**) was co-immobilised to determine the PEC activity for the reduction of aqueous protons (Fig. 1a). The resulting hybrid DSPEC photocathode produces H_2_ at moderate applied voltages with good photocurrents. Direct comparison with a corresponding NiO photocathode highlights the benefits of CuCrO_2_ and encourages the search for new DSPEC cathode materials.

## Results and discussion

### Synthesis and characterisation of CuCrO_2_

Scalable and straightforward procedures for preparation of CuCrO_2_ make it a highly accessible material, and its metal oxide character ensures that molecular species can be easily attached to the surface using anchoring groups such as phosphonic acids or carboxylic acids.^59^–^63^ In this study, CuCrO_2_ films were grown directly on ITO-coated glass following a previously established sol–gel route.^59^,^60^ In brief, a mixture of Cu(acetate)_2_·H_2_O (0.2 M), Cr(NO_3_)_3_·9H_2_O (0.2 M), and triethanolamine (0.4 M) in absolute ethanol was spin-coated on an ITO-coated glass substrate. These samples were annealed in air at 400 °C for 45 min before repeating the spin-coating and annealing steps to obtain a total of 6 layers. Post-annealing was carried out under N_2_ at 600 °C for 45 min to form the delafossite structure. NiO films (2 μm thick) were prepared for comparison using a previously reported hydrothermal growth method.^37^

CuCrO_2_ crystallises in a rhombohedral unit cell (space group *R*3*m*) and is a wide bandgap p-type semiconductor (*E*_g_ = 3.1 eV) exhibiting a low-lying valence band and high hole mobility.^63^,^64^ The structure consists of ‘infinite’ [CrO_2_] layers of edge-sharing [CrO_6_] octahedra linked by linear O–Cu–O dumbbells and the p-type conductivity stems predominantly from Cu^+^ vacancies in the crystal lattice.^65^,^66^ Favourable mixing of Cr 3d states with O 2p states increases the covalent nature of this interaction in the valence band, hence holes are more delocalised than in other corresponding delafossite structures, accounting for the intrinsic high hole mobility.^64^,^66^

**Fig. 2:**
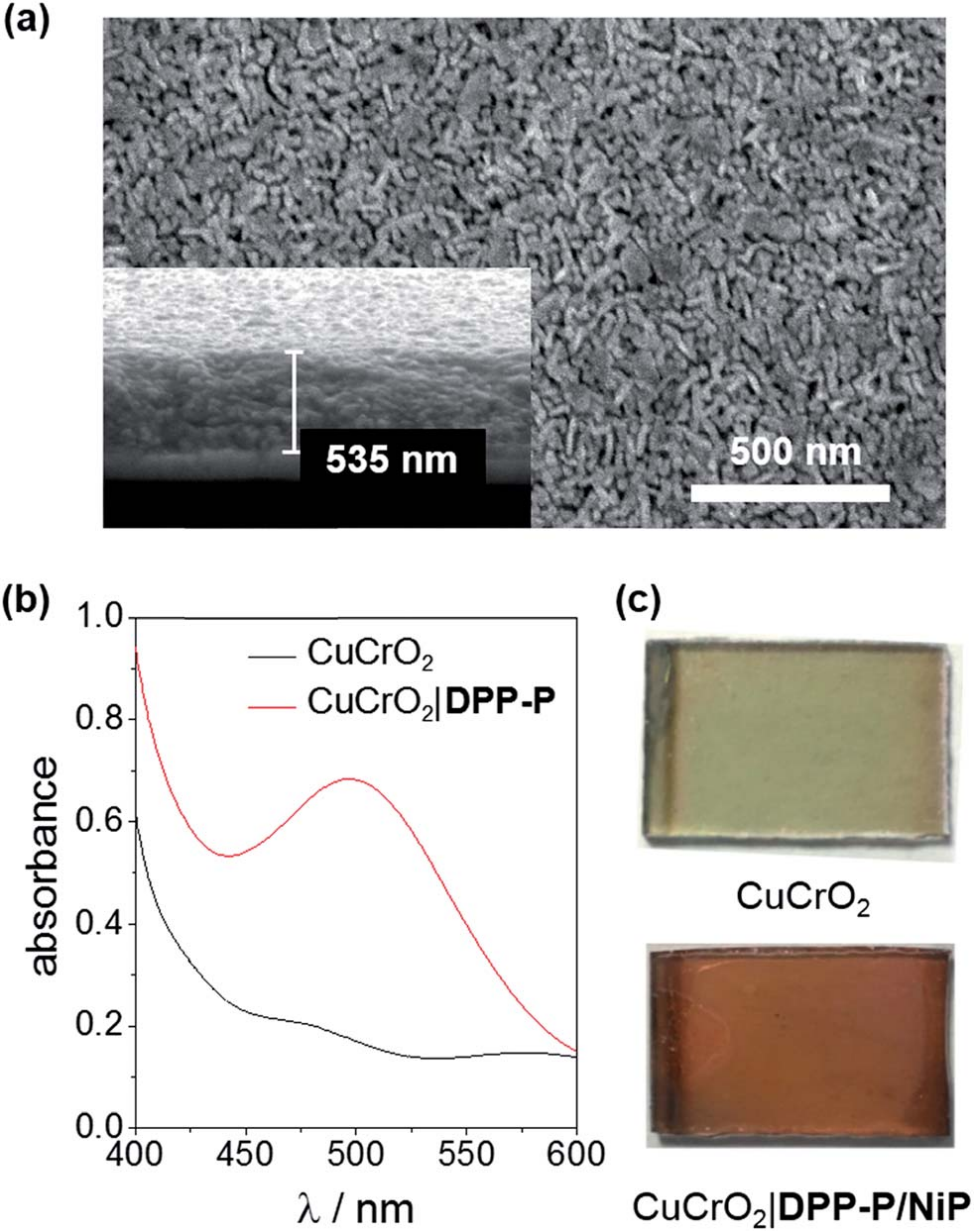
(a) Top-down and cross-sectional (inset) images of a 6-layer CuCrO_2_ electrode, (b) transmission UV-Vis spectrum of CuCrO_2_ and CuCrO_2_|**DPP-P** electrodes (ITO-glass background subtracted), (c) photographs of the as-prepared CuCrO_2_ and CuCrO_2_|**DPP-P**/**NiP** electrodes.

X-ray diffraction (XRD) analysis confirmed the rhombohedral delafossite structure for CuCrO_2_ (Fig. S1†) and scanning electron microscopy (SEM) images showed individual rods with a length of 73.3 ± 16.5 nm and thickness of 20.7 ± 3.7 nm, leaving a pore diameter of 16.7 ± 4.8 nm (Fig. 2a). The CuCrO_2_ film (resulting from 6 layers) was approximately 500 nm thick. N_2_ gas adsorption isotherms showed type IV behaviour consistent with a mesoporous material and gave a BET surface area of 25 m^2^ g^–1^ (Fig. S2†), which is similar to that obtained with other mesoporous structures.^47^ The direct bandgap of CuCrO_2_ was estimated from a Tauc plot as 3.1 eV (Fig. S3†) and the flatband potential, *E*_fb_, of +1.0 V *vs.* RHE with Mott–Schottky analysis from consecutive impedance scans (Fig. S4†). This is 0.25 V more positive than the *E*_fb_ of our NiO electrodes.^37^ See Experimental section for more details about synthesis and characterisation of the electrodes.

### Components of the molecule-loaded CuCrO_2_ photoelectrode

As dye and catalyst species, we selected **DPP-P** and **NiP** respectively, both recently synthesised in our group (Fig. 1a).^67^,^68^ For the most suitable light absorber, a dye with sufficient driving force to reduce the H_2_ evolution catalyst as well as a thermodynamically accessible reduction potential for the extraction of holes by CuCrO_2_ is required. Diketopyrrolopyrrole (DPP) chromophores have recently displayed high activity with NiO in p-DSSCs and are considered suitable candidates due to their high photostability, simple synthesis and modification, and lack of precious metal elements.^36^**DPP-P** absorbs strongly in the visible range (*ε*_496 nm_ = 2.6 × 10^4^ M^–1^ cm^–1^, DMF)^67^ and is expected to undergo reductive quenching when immobilised on a p-type semiconductor due to fast hole injection originating from the proximity and good electrical communication between the dye and semiconductor.^69^–^74^ In this pathway, the first step upon dye excitation is the reduction of DPP-P* by hole injection into the valence band of CuCrO_2_, followed by oxidation of **DPP-P^–^** by the catalyst, which ultimately performs the chemical reaction. **NiP**, a Dubois-type Ni-catalyst^75^,^76^ featuring four phosphonic acid anchoring groups, has previously demonstrated reduction of aqueous protons both in solution and when immobilised on a semiconductor surface whilst maintaining molecular integrity during photocatalysis.^5^,^6^,^67^,^68^ **DPP-P** has a reduction potential in the excited state of +1.57 V *vs.* RHE and the reduced dye has an oxidation potential of –0.7 V *vs.* RHE, thus **DPP-P^–^** can provide sufficient driving force for the reduction of **NiP** to a catalytically active state (onset of catalytic current for **NiP** = –0.21 V *vs.* RHE).^68^ The respective electrochemical potential of each component and the hole and electron transfer pathways for the fully assembled **DPP-P**/**NiP**-modified CuCrO_2_ electrode is shown in Fig. 1b and the corresponding energy diagram with possible recombination routes in Fig. S5.†

### Photoelectrochemistry of CuCrO_2_|**DPP-P**

**Fig. 3:**
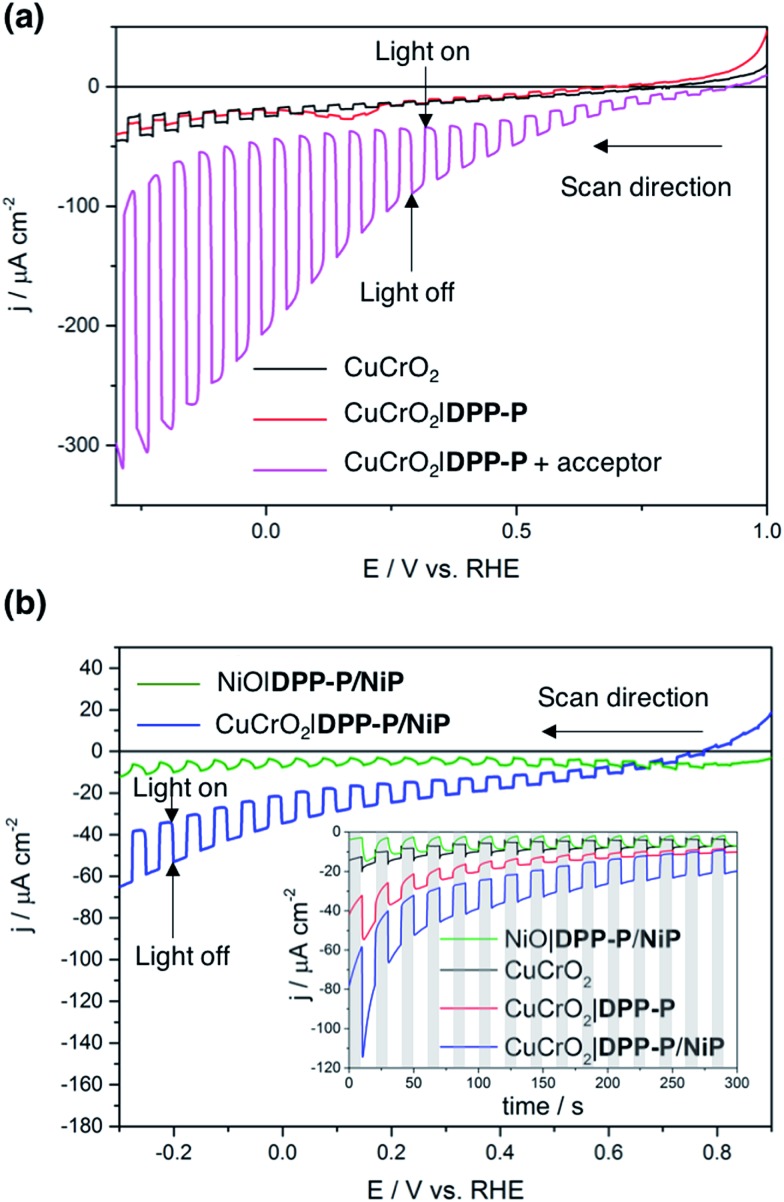
Linear sweep voltammograms under chopped light illumination of (a) CuCrO_2_ (black) and CuCrO_2_|**DPP-P** (red) electrodes, and a CuCrO_2_|**DPP-P** electrode with 5 mM DTDP acceptor in solution (magenta), (b) LSV scans of CuCrO_2_|**DPP-P**/**NiP** (blue) and NiO|**DPP-P**/**NiP** (green) electrodes along with chronoamperograms (inset) of all relevant electrode compositions. The dark chops are shown with grey lines for the chronoamperograms. All experiments were performed in aqueous Na_2_SO_4_ electrolyte solution (0.1 M) adjusted to pH 3. Illumination with 100 mW cm^–2^, AM 1.5G, with a 420 nm cutoff filter at room temperature. An active electrode area of 0.25 cm^2^ was used with a scan rate of 5 mV s^–1^ for voltammograms.

To evaluate the compatibility of **DPP-P** with CuCrO_2_ and to ensure this interface could function without the kinetic limitations imposed by immobilisation of a molecular catalyst, PEC measurements were conducted on dye-sensitised electrodes in the presence of a soluble electron acceptor. These photocathodes were prepared by soaking CuCrO_2_ electrodes in a **DPP-P** solution (1 mM, DMF) for 15 h. The UV-Vis spectrum of the electrodes with immobilised **DPP-P** displays an absorption maximum at approximately 500 nm, consistent with the electronic transition of the free dye (Fig. 2b and c).^67^ Linear sweep voltammetry (LSV) and chronoamperometry experiments were carried out in an aqueous Na_2_SO_4_ electrolyte solution (0.1 M, pH 3) at room temperature in a N_2_-purged one-compartment three-electrode electrochemical cell using a Pt counter electrode and a Ag/AgCl/KCl_sat_ reference electrode. UV-filtered simulated solar light was used for all PEC measurements (100 mW cm^–2^, AM 1.5G, *λ* > 420 nm). In control experiments without the acceptor, the bare CuCrO_2_ electrodes displayed a small cathodic dark current, which has previously been attributed to the reduction of Cu^2+^ impurities to Cu^+^ with oxygen deintercalation (Fig. 3a).^77^ Irradiation of the unmodified and **DPP-P** modified electrodes resulted in only minor photocurrents without a soluble acceptor (|*j*| < 3 μA cm^–2^, 0.0 V *vs.* RHE) (Fig. 3a).

Addition of the electron acceptor 4,4′-dithiodipyridine (DTDP, 5 mM) in the electrolyte solution allows for estimation of a maximal attainable photocurrent as DTDP is known to be easily reduced in solution (*E*_red,DTDP_ = –0.06 V *vs.* RHE).^37^ The electron acceptor allows the photoreduced dye to dispose of photo-electrons and to regenerate the ground state, thereby limiting the effects of reductive dye decomposition and charge recombination, and dramatically enhancing the photocathodic response for CuCrO_2_|**DPP-P**. An absolute photocurrent response of ≈160 μA cm^–2^ (0.0 V *vs.* RHE, Fig. 3a) was observed, which indicates efficient light-induced hole injection from the dye to the valence band of CuCrO_2_ with reduction of the acceptor by **DPP-P^–^**. For comparison, a NiO electrode sensitised in the same manner displayed a lower maximum photocurrent (|*j*| ≈ 80 μA cm^–2^, 0.0 V *vs.* RHE), suggesting lower susceptibility to recombination between the reduced dye and holes in CuCrO_2_ (Fig. S6†). Thus, **DPP-P** displays excellent electronic communication with CuCrO_2_, which suggests that co-anchoring of a catalyst could be a viable approach to exploit the reductive power of **DPP-P^–^** for solar H_2_ production.

### Photoelectrochemistry with CuCrO_2_|**DPP-P**/**NiP**

Catalyst and dye molecules were co-immobilised on CuCrO_2_ electrodes through soaking in a solution of **NiP** (0.5 mM) and **DPP-P** (1 mM), in DMF for 15 h. The loading of **DPP-P** was quantified by UV-Vis spectroscopy following desorption in alkaline solution and the amount of immobilised **NiP** determined using inductively coupled plasma optical emission spectroscopy (ICP-OES) measurements. This resulted in a 2 : 1 ratio of dye to catalyst on the electrodes (Table S1†). Co-immobilisation of **NiP** and **DPP-P** on CuCrO_2_ resulted in a five-fold enhancement in photocurrent compared to the bare electrode (|*j*| = 15.1 μA cm^–2^, 0.0 V *vs.* RHE) (Fig. 3b). This increased response is attributed to the ability of **DPP-P^–^** to reduce **NiP** and ultimately protons.^67^ This is supported by the incident photon-to-current efficiency (IPCE) spectrum, which displays a maximum photocurrent at the same wavelength as the absorption maximum of **DPP-P** (*λ*_max_ = 500 nm, Fig. S7†). For comparison, CuCrO_2_ electrodes showed low efficiency and no peak at this wavelength, demonstrating the essential role of the sensitiser.

H_2_ generation was studied using controlled potential photoelectrolysis (CPPE) under constant light illumination with an applied potential of 0.0 V *vs.* RHE. The CPPE trace of the CuCrO_2_|**DPP-P**/**NiP** electrode showed high stability over a 2 hour period (Fig. S8†) with 94 ± 10 nmol of H_2_ generated, corresponding to a turnover number of the **NiP** catalyst (TON_cat_) of 126 ± 13 and a faradaic efficiency (FE) of 34 ± 8%. Possible explanations for the modest FE are the dark current originating from Cu^2+^ reduction and oxygen deintercalation,^77^ as well as capacitive currents due to the mesoporous structure or from electrons trapped in surface states.^56^,^78^–^80^ The FE is lowered by probable photobleaching/decomposition and desorption of the dye species, and is overall comparable to previously reported dye-sensitised photocathodes (Table 1). Control experiments without dye (CuCrO_2_|**NiP**) or catalyst (CuCrO_2_|**DPP-P**) produced no detectable hydrogen, confirming that the full assembly is required for catalysis. A comparable NiO|**DPP-P**/**NiP** electrode modified in the same manner only yielded 35 ± 2 nmol of H_2_ after 2 hours, with a FE of 31 ± 8%, demonstrating the superior performance (2–3 times) of CuCrO_2_ (Table 1). Accurate quantification of the Ni-catalyst loading on NiO was not possible by ICP-OES (same element in catalyst and substrate) or by UV-Vis spectroscopy following desorption (low molar absorption of **NiP**).

**Table 1:** Dye-sensitised photocathodes with immobilised molecular catalysts for proton reduction in aqueous solution

Substrate	Dye	Catalyst	pH	Electrolyte solution	|*j*|/μA cm^–2^@*E*_app_/V *vs.* RHE	Faradaic efficiency	TON_cat_	Reference
CuCrO_2_	**DPP-P**	**NiP**	3	Na_2_SO_4_	15@0.00	34 ± 8%	126 ± 13.3 (2 h)	This work
NiO	**DPP-P**	**NiP**	3	Na_2_SO_4_	5.8@0.00	31 ± 8%	n. r.^*a*^	This work
NiO	Ru complex (**RuP3**)	**NiP**	3	Na_2_SO_4_	∼10@0.30	8.6 ± 2.3%	≈1 (2 h)^*b*^	37
NiO	Dyad organic dye-cobalt diimine-dioxime		5.5	MES/NaCl	15@0.14	9.5%	≈4 (2 h)^*b*^	18
NiO	Supramolecular Ru complex-cobaloxime assembly		7	Phosphate	8@0.51	68%	n. r.^*a*^	40
NiO	Organic dye (**P1**)	Cobaloxime	7	Phosphate	∼5@0.21	68%	n. r.^*a*^	39
NiO	Ru complex (**RuP**)	Cobaloxime (**CoHEC**)	7	Phosphate	13@0.21	n. r.^*a*^	n. r.^*a*^	41
NiO	Coumarine **C343**	Fe_2_(CO)_6_(bdt)	4.5	Acetate	∼10@0.16	50%	≤3 (18 min)	38
NiO	CdSe	Cobaloxime	6.8	Na_2_SO_4_	100@0.40	81%	n. r.^*a*^	42
ITO	Supramolecular Ru complex-(**RuP2**)-**NiP** assembly		5.1	MES	56@0.05	53 ± 5%	≈16 (3 h)^*b*^	43
NiKO|NiCuO|ioITO	Supramolecular Ru complex (**RuP2**)-**NiP** assembly		5.0	MES/KCl	∼59@0.05	∼90%	≈20 (2 h)	44

^*a*^n. r.: not reported.

^*b*^Calculated from the reference.

Post-electrolysis characterisation of CuCrO_2_|**DPP**/**NiP** electrodes using ICP-OES showed that the amount of **NiP** retained on the surface after 2 h of CPPE was 54% of the initial loading (Table S1†). This is in part due to the relatively low surface area exhibited by the delafossite particulates (25 m^2^ g^–1^), which accounts for low loadings of catalyst and dye, and allows for their easy desorption into the media. Nanostructuring of the surface would ensure higher loadings of dye and catalyst species, enhancing both stability and activity in the future. Alternate methods such as atomic layer deposition (ALD)^52^,^81^–^83^ or polymeric assembly^84^–^87^ could also be employed as additional stabilisation methods.

### Comparison with state-of-the-art

Limited improvements in photocathode development for DSPEC proton reduction are largely due to p-type materials with low performance. Since the first report in 1999 towards p-type DSSC, dye-sensitised NiO electrodes have generated a range of beneficial research on dye architecture and electrolyte composition.^48^,^88^,^89^ Despite this, their performance remains significantly lower than their n-type counterparts, highlighting the limitations of NiO and the need for a better alternative. Table 1 highlights relevant examples as a comparison for our system.

The TON_cat_ is a good measure of catalytic activity for a molecular catalyst-based system but remains unreported in most cases. A TON_cat_ > 125 after 2 h for our CuCrO_2_ system in water compares favourably with the currently highest reported value of ≈20 for a NiO DSPEC photocathode.^43^

With **NiP** as the catalyst, an ITO electrode produced higher photocurrents and more H_2_,^44^ but PEC activity has only been demonstrated for an applied potential of +0.05 V *vs.* RHE. CuCrO_2_ allows for a much higher working voltage due to the onset potential being situated at +0.75 V *vs.* RHE and therefore shows greater suitability for energy storage and implementation in tandem DSPEC cells. This photocurrent onset is also more favourable than other commonly used narrow bandgap p-type semiconductors such as GaP,^90^,^91^ and p-Si,^5^,^92^ highlighting the benefits of moving to dye-sensitised systems for H_2_ generation.

CdSe-sensitised NiO produces the highest amount of H_2_ of these electrodes over the duration of 2 hours of CPPE,^42^ but a large portion of the photocurrent stems from the bare quantum dots. Despite this, sensitisation with quantum dot species is a viable approach to further enhance the H_2_ producing capability of a CuCrO_2_-based photocathode in the future. In comparing these properties, it is clear that material alteration can have a great influence on activity, and that transferring from NiO to CuCrO_2_ has advantages for DSPEC applications.

## Conclusions

We have introduced CuCrO_2_ co-sensitised with an organic dye (**DPP-P**) and molecular catalyst (**NiP**) for DSPEC H_2_ generation under aqueous conditions. CuCrO_2_|**DPP-P**/**NiP** showed a photocurrent onset at +0.75 V *vs.* RHE and a photocurrent density of 15 μA cm^–2^ at 0.0 V *vs.* RHE with a TON_cat_ of 126 ± 13 achieved in controlled potential photoelectrolysis under UV-filtered simulated solar light irradiation. The molecule-loaded delafossite electrode therefore surpasses the performance of benchmark NiO electrodes in side-by-side comparison. We also show that the phosphonated organic DPP dye allows for high performance in aqueous conditions on an electrode and is able to electronically cooperate with **NiP**, which enabled us to assemble a fully precious metal-free DSPEC photocathode. The photocathode displays a higher photovoltage than other current state-of-the-art materials such as p-Si and GaP, making it well suited for coupling with a photoanode in tandem water splitting. Co-immobilisation of a dye and a CO_2_ reduction catalyst on this p-type semiconductor may allow photocathodic production of carbon based fuels and chemical feedstocks.

The synthesis of CuCrO_2_ by sol–gel techniques is straightforward and scalable. Nanostructuring would enhance the molecular loading and provide another avenue to increase photocurrents and the H_2_ producing capability of the photocathode. Material alteration, for example through Mg^2+^ doping,^62^ could also improve the activity by further enhancing conductivity and therefore charge extraction through the film. Other methods to improve the separation between catalyst and the delafossite surface would also enhance the efficiency by reducing charge recombination.^37^ This work demonstrates the benefit of adopting new delafossite structures as p-type semiconductors for solar fuel generation.

## Experimental section

### Materials and methods

**NiP** 68 and **DPP-P**^67^ were synthesised according to previously reported methods. Cu(acetate)_2_·H_2_O (ACROS Organics, ACS reagent), Cr(NO_3_)_3_·9H_2_O (Sigma-Aldrich, ≥99%), and triethanolamine (Sigma-Aldrich, ≥99.5%) were used to prepare CuCrO_2_. ITO-coated glass substrates (Vision Tek Systems Ltd., *R* = 12 Ω cm^–2^, thickness = 1.1 mm) were cut into 3 × 3 cm^2^ squares and scored into 1 × 1.5 cm^2^ divisions before cleaning. Milli-Q® H_2_O (*R* > 18.2 MΩ cm) was used for all electrochemical and analytical measurements. DTDP (Sigma-Aldrich, 98%) was used as an electron acceptor at a concentration of 5 mM. Addition of DTDP resulted in a change in pH of the electrolyte solution from 3 to 4.6.

### Preparation of CuCrO_2_ electrodes

ITO-coated glass was cleaned through successive sonication in isopropanol, ethanol, and acetone for 15 min each, followed by drying at 100 °C in air before use. A mixture of Cu(acetate)_2_·H_2_O (0.2 M), and triethanolamine (0.4 M) in absolute ethanol was stirred for 1 h before addition of Cr(NO_3_)_3_·9H_2_O (0.2 M). This solution was kept stirring for 15 h before being spin-coated on the ITO-glass slides (Laurell WS-650MZ spin coater, 1500 rpm, 15 s, 3000 rpm s^–1^ acceleration, 0.4 mL volume). The slides were annealed in air to 400 °C for 45 min with a ramp rate of 10 °C min^–1^ in a chamber furnace (Carbolite Gero). These steps were repeated to form 6 layers. The final annealing step involved heating in a N_2_ atmosphere to 600 °C for 45 min with a ramp rate of 5 °C min^–1^ using a tube furnace fitted with a quartz tube, end seals, and insulation plugs (Carbolite Gero). The electrodes were left to cool to room temperature and used as-prepared without any additional treatment.

### Material characterisation

XRD measurements were conducted using a PANalytical BV X'Pert Pro X-ray diffractometer. SEM images were taken using a FEI Phillips XL30 sFEG microscope. UV-Vis absorption spectra were obtained using a Varian Cary 50 spectrophotometer in transmission mode.

### N_2_ gas adsorption measurements

Adsorption isotherms were carried out using a Micromeritics 3 Flex (Micromiretics, Norcross, GA, USA) with N_2_ as the adsorbate. Samples were prepared on glass slides then scraped from the surface. Degassing for 10 h at 110 °C was required prior to measurements, which were carried out in liquid N_2_. The BET specific surface area was obtained by fitting N_2_ isotherms using the Microactive software.

### Mott–Schottky analysis

Electrochemical impedance spectroscopy (EIS) measurements were conducted using an IviumStat potentiostat at 25 °C using a 3-necked round-bottomed flask under dark conditions. A three-electrode setup using a Pt mesh counter, Ag/AgCl/KCl_sat_ reference, and a CuCrO_2_ working electrode (0.25 cm^2^ active area) was used with an electrolyte solution of Na_2_SO_4_ (0.1 M, pH 3). The frequency range was 10 kHz to 0.01 Hz, with an excitation voltage of 10 mV. Nyquist plots obtained in the potential range 1.1 V to 0.3 V *vs.* RHE (15 mV step) were fitted using ZView® (Scribner Associates Inc.) to a Randles circuit (inset Fig. S4†) to obtain interfacial capacitance (*C*_sc_) values. The Mott–Schottky equation, 
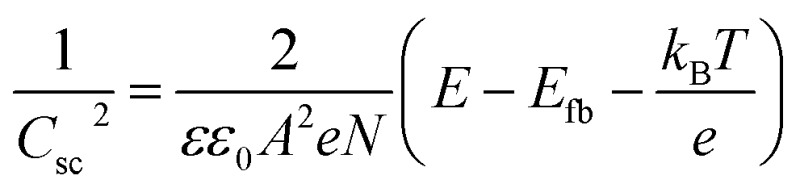
, was used to obtain an estimate of the flatband potential through a plot of 1/*C*_sc_^2^ against the applied potential. A negative slope indicated p-type character and the *x*-intercept is equal to *E*_fb_ + *k*_B_*T*/*e*.^37^

### Electrochemical measurements

Cyclic voltammetry was used to determine the reduction potential of the **DPP-P** dye, *E*_(S/S^–^)_, from the half-wave potential. This was performed in a 3-electrode setup with a glassy carbon working electrode, Pt-mesh counter electrode, and a Ag/AgCl/KCl_sat_ reference electrode with a scan rate of 50 mV s^–1^. The electrolyte solution consisted of tetrabutylammonium tetrafluoroborate (0.1 M) in dry DMF with the addition of **DPP-P** (around 0.1 M). Addition of the *E*_00_ to *E*_(S/S^–^)_ provides an estimate for the excited state reduction potential, *E*_(S*/S^–^)_.

### Modification of electrodes with dye and catalyst species

Molecular species were co-immobilised through soaking in a bath consisting of **DPP-P** (1 mM) and **NiP** (0.5 mM) in DMF for 15 h. For CuCrO_2_|**DPP-P** and CuCrO_2_|**NiP** electrodes the concentration was 1 mM but all other conditions kept the same. All electrodes were rinsed with DMF and H_2_O then dried in air and stored in the dark before use.

### Quantification of loaded **DPP-P** and **NiP**

**DPP-P** was desorbed from CuCrO_2_|**DPP-P**/**NiP** electrodes using a solution of 0.1 M tetrabutylammonium hydroxide 30-hydrate in DMF (1 mL) and the absorption at 500 nm was determined using UV-Vis spectroscopy. A calibration curve was used to fit values and determine the loading for 4 different electrodes. **NiP** was quantified by ICP-OES after digestion of CuCrO_2_|**DPP-P**/**NiP** electrodes (1 cm^2^ film area) in aqueous HNO_3_ (70%, 1 mL) overnight and dilution to 10% v/v with MilliQ® water. CuCrO_2_|**DPP-P**/**NiP** electrodes pre- and post-electrolysis were analysed along with blanks for nitric acid, CuCrO_2_, and CuCrO_2_|**DPP-P** in triplicate. Errors represent standard deviation from the mean.^37^

### PEC measurements

Photoelectrochemical measurements were carried out using an Ivium CompactStat potentiostat in a one-compartment three-necked custom made cell equipped with a flat borosilicate glass window. A three-electrode setup was used with a Pt-counter electrode, a Ag/AgCl/KCl_sat_ reference, and the working electrode consisted of the CuCrO_2_ platform with an illuminated area of 0.25 cm^2^ confined using electrical tape. All measurements were conducted using aqueous Na_2_SO_4_ electrolyte solution (0.1 M, pH 3) and the cell was purged with N_2_ for 15 min prior to experiments. Frontside illumination was used for all experiments using a calibrated Newport Oriel solar light simulator (150 W, 100 mW cm^–2^, AM 1.5G) fitted with a UQG Optics UV Filter (*λ* > 420 nm) and IR water filter.

CPPE experiments were carried out in a custom two-compartment airtight electrochemical cell separated by a Nafion membrane and featuring a flat quartz glass window. The volume of electrolyte solution in the working compartment was 12 mL with a gas headspace of 5 mL while the counter compartment consisted of 4.5 mL solution and a 3.5 mL headspace. Prior to electrolysis, the gas headspace was purged for 30 min with 2% CH_4_ in N_2_. An Agilent 7890A series gas chromatograph with a 5 Å molecular sieve column and a thermal conductivity detector was used to quantify the amount of H_2_ produced. The oven temperature was kept constant at 45 °C and the flow rate was 3 mL min^–1^. The partial pressure of H_2_ was calculated to account for dissolved H_2_ and this was added to the overall amount of hydrogen generated to obtain the faradaic efficiency. All CPPE experiments were carried out in triplicate with an applied potential of 0.0 V *vs.* RHE.

### IPCE measurements

IPCE spectra were recorded in a N_2_-purged three-necked one-compartment custom cell with a flat borosilicate glass window. A three-electrode setup with Pt counter, Ag/AgCl/KCl_sat_ reference, and working electrode was used with pH 3 Na_2_SO_4_ electrolyte solution (0.1 M). Monochromatic light was provided using a 300 W Xenon lamp solar light simulator coupled to a monochromator (MSH300, LOT Quantum design) and the intensity calibrated to 0.8 mW cm^–2^ for each wavelength. The potential was maintained at 0.0 V *vs.* RHE for all wavelengths and photocurrents were recorded in triplicate with different electrodes (0.25 cm^2^ active area) for both CuCrO_2_ and CuCrO_2_|**DPP-P**/**NiP** arrangements.

## Conflicts of interest

There are no conflicts to declare.

## Supplementary Material

Supplementary information
