# Supplementary material for: Solar H2 generation in water with a CuCrO2 photocathode modified with an organic dye and molecular Ni catalyst
Source: Chem Sci. 2017 Nov 27;9(6):1439–47. doi: 10.1039/c7sc04476c (PMC5875021; doi:10.1039/c7sc04476c)
Supplement: Supplementary file 1 [file SC-009-C7SC04476C-s001.pdf]

Electronic Supplementary Material (ESI) for Chemical Science.
This journal is © The Royal Society of Chemistry 2017

Electronic Supporting Information

Solar H_2 generation in water with a $CuCrO_2$ photocathode modified with an organic dye and molecular Ni catalyst

Charles E. Creissen[a], Julien Warnan[a], Erwin Reisner[a]*

[a]Christian Doppler Laboratory for Sustainable SynGas Chemistry, Lensfield Road, CB2 1EW, Cambrigde, UK.

*Correspondence to: reisner@ch.cam.ac.uk

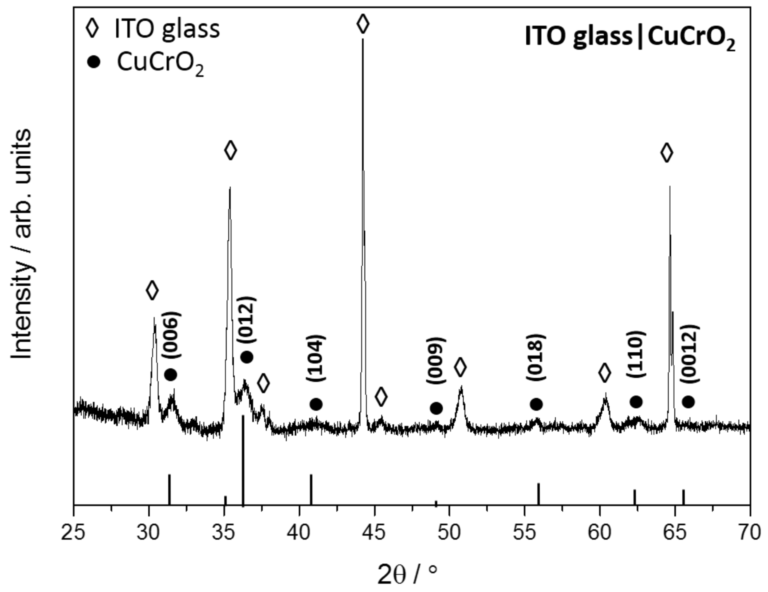

Figure S1. XRD pattern for a $CuCrO_2$ film grown on an ITO-coated glass substrate. Diamond symbols represent peaks for the ITO glass substrate and dots correspond to $CuCrO_2$ (ICSD collection code 026676).

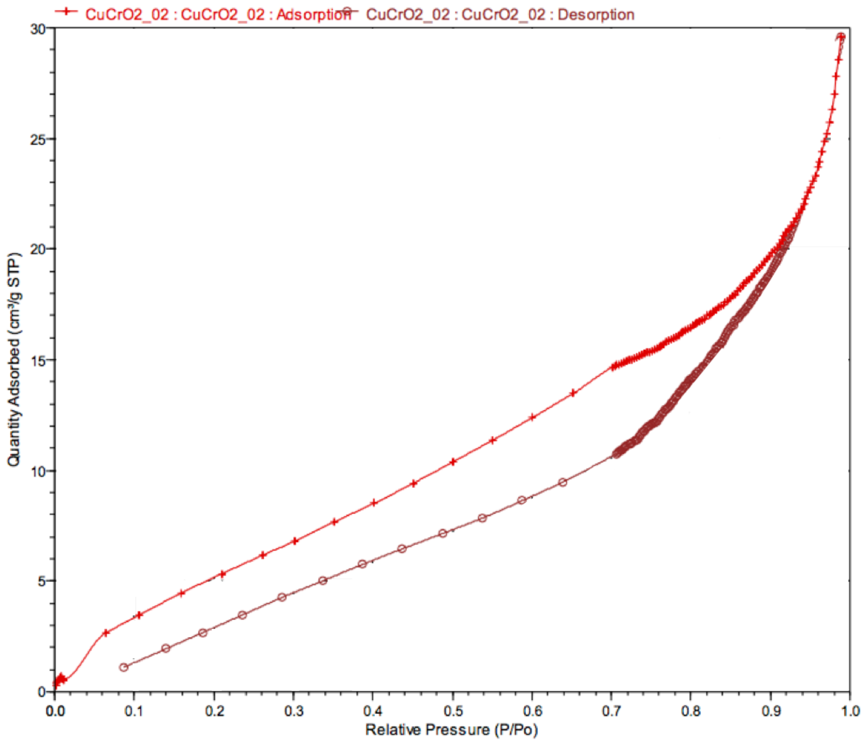

Figure S2. N_2 adsorption isotherm obtained using $CuCrO_2$ powder scraped from the surface of electrodes used to obtain BET specific surface area.

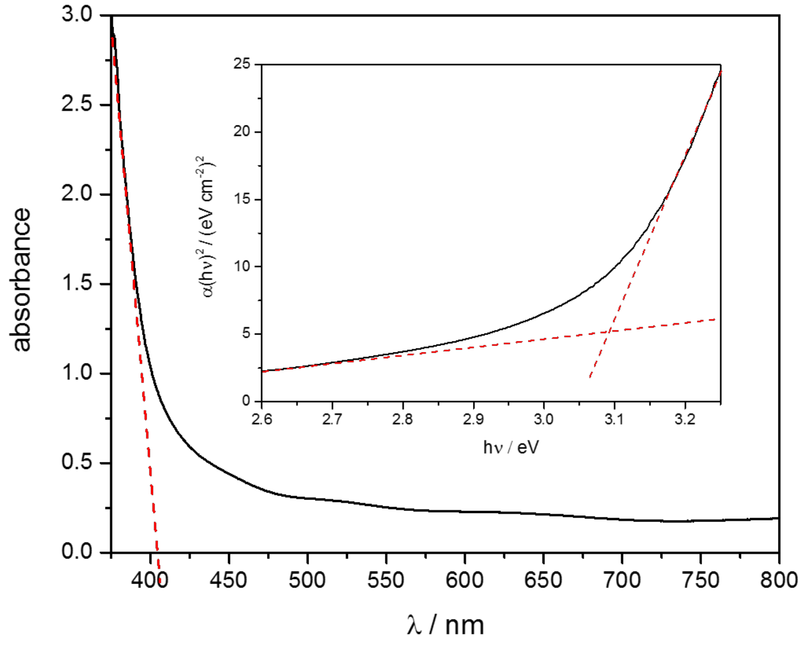

Figure S3. UV-vis spectrum and Tauc Plot (inset) for a $CuCrO_2$ film grown directly on an ITO-coated glass substrate to show the direct bandgap. ITO-glass background was subtracted. Spectra were obtained using a Varian Cary 50 spectrophotometer in transmission mode.

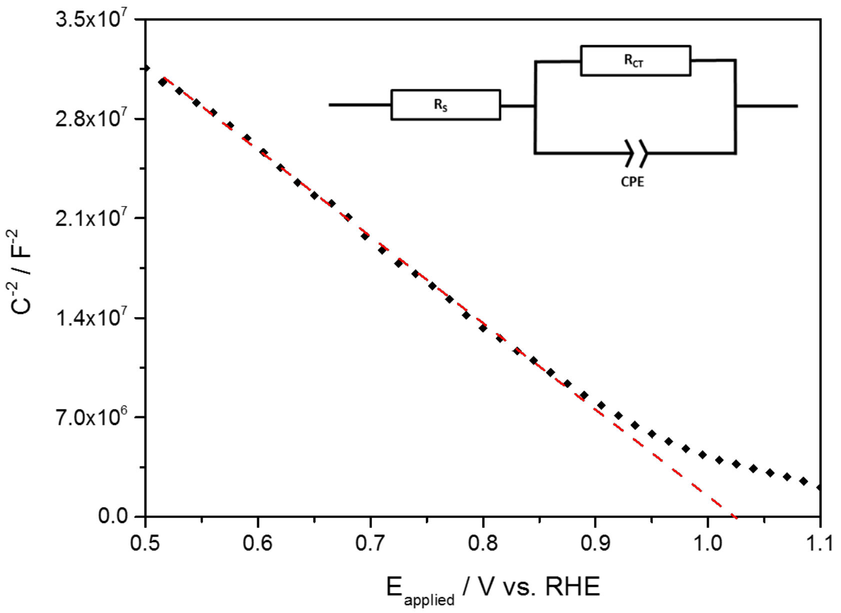

Figure S4. Mott-Schottky plot for a $CuCrO_2$ electrode in Na_2SO_4 (0.1 M, pH 3) at room temperature. Frequency range 10 kHz to 0.1 Hz with 10 mV excitation voltage. The Randles circuit shown (inset) was used to fit Nyquist plots obtained at each potential in the ZView® software.

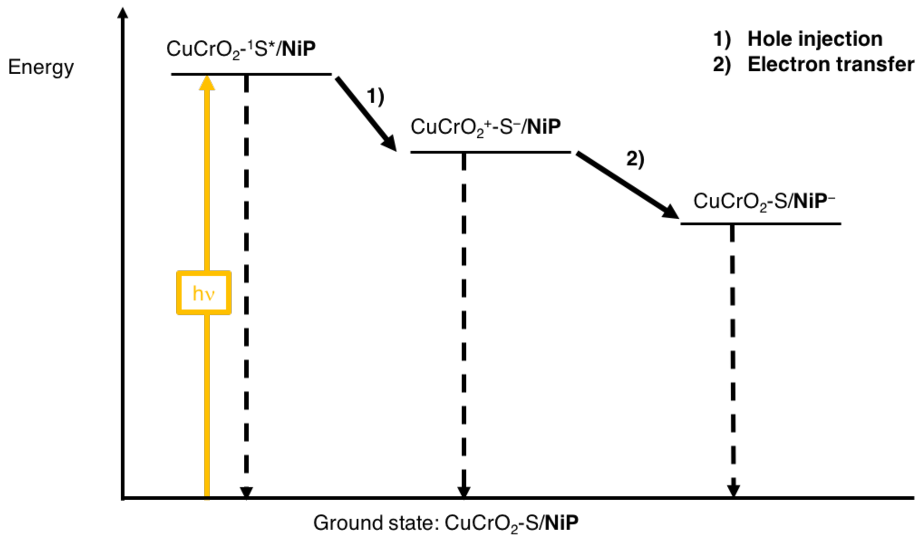

Figure S5. Simplified energy diagram showing hole and electron transfer alongside possible recombination routes (dashed arrows). The catalytic onset potential corresponds to the second reduction of **NiP** but is omitted here for simplicity. S: Sensitiser.

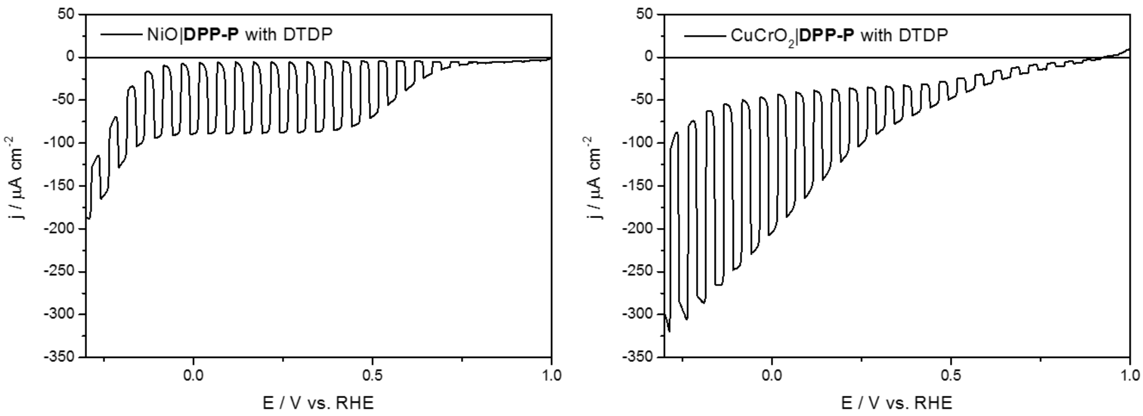

Figure S6. Linear sweep voltammograms for NiO|**DPP-P** (left) and $CuCrO_2$|**DPP-P** (right) electrodes with DTDP acceptor (5 mM) added to Na_2SO_4 (0.1 M), pH 4.56. Illumination with 100 mW cm^{-2}, AM 1.5 G, with a 420 nm cutoff filter and an active electrode area of 0.25 cm^2. A scan rate of 5 mV s^{-1} was used and experiments were carried out at room temperature.

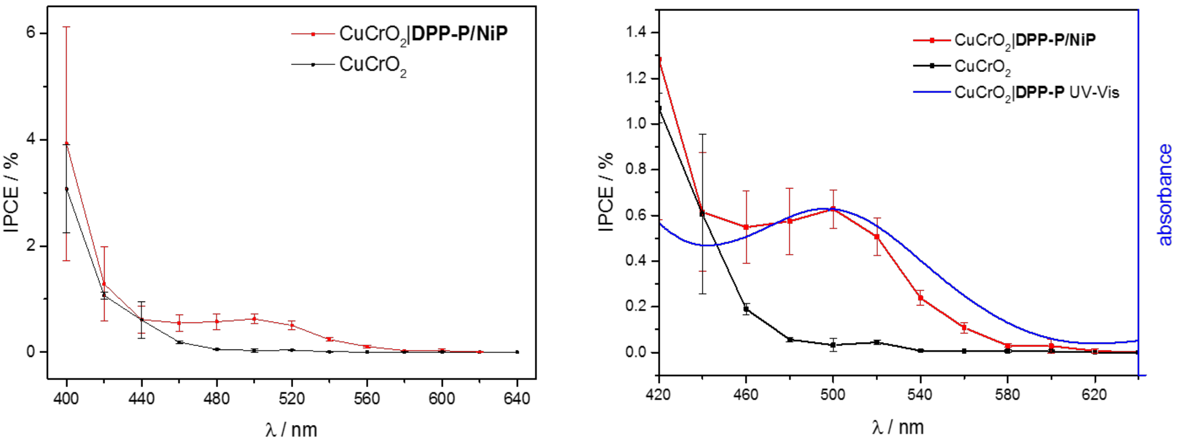

Figure S7. IPCE spectra for $CuCrO_2$ and $CuCrO_2$|**DPP-P/NiP** (left) and the same IPCE spectra with the UV-Vis of $CuCrO_2$|**DPP-P** overlaid (right). All values were recorded in Na_2SO_4 (0.1 M, pH 3) at room temperature with an applied potential of 0.0 V vs. RHE and light intensity maintained at 0.8 mW cm^{-2}.

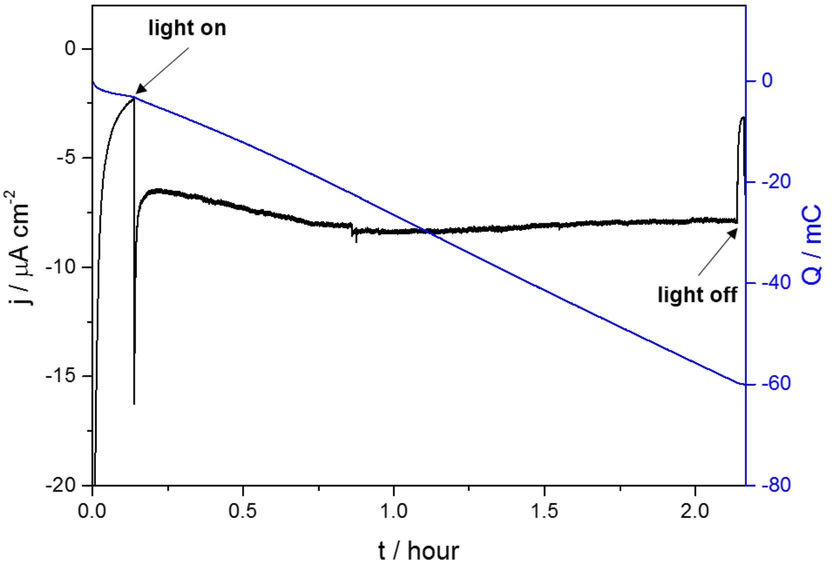

Figure S8. CPPE trace of a $CuCrO_2$|**DPP-P/NiP** electrode held at 0.0 V vs. RHE in Na_2SO_4 electrolyte solution (0.1 M) adjusted to pH 3. Illumination with 100 mW cm^{-2}, AM 1.5 G with a 420 nm cutoff filter and an active electrode area of 1 cm^2. The cell was maintained at room temperature.

Table S1. Dye and catalyst loadings for the $CuCrO_2$|**DPP-P/NiP** with the specific surface area of $CuCrO_2$.

**Electrode**	**NiP / nmol cm^{-2}**	**NiP post-electrolysis/ nmol cm^{-2}**	**DPP-P / nmol cm^{-2}**	**Specific Surface Area / m^2 g^{-1}**
$CuCrO_2$|**DPP-P/NiP**	0.75 ± 0.40	0.41 ± 0.30	2.55 ± 0.66	24.9

End of Supporting Information
